# Comorbid ADHD and Bipolar Disorder - An Update

**DOI:** 10.1177/10398562251351448

**Published:** 2025-06-13

**Authors:** Gordon Parker

**Affiliations:** Discipline of Psychiatry & Mental Health, School of Clinical Medicine, 7800 University of New South Wales, Sydney, NSW, Australia

**Keywords:** attention-deficit disorder, attention-deficit-hyperactivity disorder, bipolar disorder, comorbidity

## Abstract

**Objective:**

To note the strong comorbid link between ADHD and the bipolar disorders, consider candidate mechanisms and nominate a preferred management approach.

**Conclusions:**

Comorbid ADHD and bipolar disorder is distinctly higher than expected if they are independent conditions and their linkage is capable of multiple explanations. In managing such states a sequential approach is favoured, with the bipolar condition being brought under control first before initiating any stimulant medication for the ADHD.

This paper seeks to draw clinician attention to the high rate of ADD (attention-deficit disorder) or ADHD (attention-deficit hyperactivity disorder) in patients with a bipolar disorder. While long noted in the literature and overviewed earlier by this author,^1^ its commonality argues for diagnostic pursuit by clinicians when seemingly only one condition has been nominated or identified.

ADHD prevalence rates in Australia have been quantified as in the order of 6%–10% for children and adolescents and 2%–6% for adults,^2^ while the lifetime rate for a bipolar disorder in Australians was estimated to be 3.8% in the Australian Bureau of Statistics 2020-21 survey.^3^ Assuming respective prevalence rates of 6% and 4%, the prevalence rate of having both conditions should then be in the order of 0.24% if they are independent.

Empirical studies report distinctly higher rates. For example, Salvi et al.^4^ quantified comorbid rates ranging from 9.5% to 36% across relevant studies and thus far in excess of what might be expected if they were truly independent conditions. They also reported data from several studies evaluating the chance of developing a bipolar disorder in those with ADHD and with the rates ranging from a four-fold to a ten-fold increase in the emergence of a bipolar disorder in young adults (and similar for both the bipolar I or bipolar II disorders). Schiweck et al.^5^ undertook a systematic review of 71 relevant studies involving nearly six hundred and fifty thousand participants. They quantified that 7.9% of adults with ADHD were diagnosed with a bipolar disorder and, of those with a bipolar disorder, 17.1% had an ADHD diagnosis – with bipolar disorder type again not influencing rates.

While comorbidity in psychiatric conditions in general has been described and quantified as ‘pervasive’^6^ one link is the focus in this paper. The data in relation to ADHD and bipolar disorder invite speculation as to possible explanations for their high coterminous rate, possible underlying mechanisms and, of key importance here, management considerations.

It could reflect a referral artefact. Bartoli et al.^7^ reported a systematic review and meta-analysis of 43 studies involving those with the ADHD and bipolar conditions (compared to those with a bipolar condition alone). Subjects were more likely to have several anxiety disorders, eating disorders, alcohol and substance use disorders, and an antisocial personality disorder, and to have experienced more mood episodes at both poles and more mixed episodes and were more likely to have attempted suicide. As most of their contributing studies were of psychiatric patients rather than community samples, they may have had more severe symptomatic states and/or experienced greater impairment, and thus sought or been referred for professional assistance and so effecting a bias.

Turning to potential linkage determinants, Comparelli et al.^8^ referenced several supportive familial and genetic studies to suggest that the two conditions might simply reflect a single ADHD-bipolar disorder entity, although most researchers argue for two independent but coterminous conditions. Klassen et al.^9^ suggested that such co-segregation could reflect (i) chance, (ii) an artefact of overlapping criteria, (iii) a common diathesis, and (iv) preceding ADHD symptoms reflecting pre-pubertal expression of a bipolar disorder prior to formal presentation. The rich field studies (with several noted earlier) reject chance as an explanation.

The second explanation implies ‘false comorbidity’ in play. While there are some hypo/manic symptoms that might be shared with ADHD (such as excessive talking, distractibility, physical restlessness, and attentional disturbance), Klassen and colleagues^9^ reported one study indicating that this explanation cannot be sustained as all explanatory. The fourth explanation again offers a ‘false comorbidity’ model but does not account for the high comorbidity rates in studies measuring each condition with some precision.

The third explanation of a shared diathesis (be it reflecting a biological or environmental factor) has been widely considered. In relation to a genetic contribution, Faraone et al.^10^ undertook a meta-analysis of relevant family-based studies and quantified that relatives of probands with a bipolar disorder had a higher chance of having ADHD and, in addition, that bipolar I disorder was more prevalent in relatives of ADHD probands. Comparelli et al.^8^ also overviewed studies quantifying that relatives of patients with either condition had a greater chance of having the other condition and with genetic studies quantifying up to 33 shared loci and so supporting a genetic contribution. However, a Consortium^11^ genome-wide analysis quantified only a small (albeit significant) genetic correlation and instead supported gene by environment interactions as a stronger candidate explanation.

Several papers have speculated on a shared environmental diathesis. For example, Schiweck et al.^7^ listed premature birth, low birth weight, maternal substance abuse, maternal stress during the first trimester of pregnancy, and childhood adversity being over-represented in those with both conditions but emphasized the non-specificity of such findings.

There may be shared biological factors in play. For example, Bartoli et al.^12^ noted evidence of dysfunction of dopamine in the brain reward circuitry in each condition and so potentially providing a link.

Any diathesis factor could act as a high-order shared factor or at the outcome level – whereby the presence of one condition increases the chance of the other condition emerging. As ADHD is generally defined as having an onset in early childhood and the bipolar disorders rarely have a pre-pubertal onset, the most likely sequence would be of ADHD increasing the risk of a bipolar disorder emerging. In a sub-set of 14 studies reporting age of onset data, Schiweck and colleagues^7^ quantified an earlier age of onset (of some 4 years) of bipolar disorder in those with comorbid ADHD compared to those without ADHD. Such a finding is relevant in considering determinants. For example, ADHD-induced neural dysfunction may contribute to the onset of the bipolar condition in those so disposed.

The prescription of a stimulant medication to an individual with ADHD could induce a bipolar disorder in those so predisposed – another sequencing model. Or, alternately, induce a bipolar III condition (where the stimulant acts akin to other known ‘induction’ drugs – such as steroids and antidepressants) in inducing iatrogenic hypo/manic states. There would appear to be only one salient study addressing this possibility. In a Taiwanese case register-based study,^13^ 145,000 children newly diagnosed with ADHD and treated with methylphenidate were followed for over a decade. The participants, however, had a slightly lower risk of new-onset bipolar disorder compared to controls, and thus rejecting such an explanation.

While it would appear highly unlikely that a bipolar disorder might induce the onset of an ADHD condition, clinical observation identifies patients with no evidence of ADHD in childhood or adolescence having onset of a bipolar disorder in adolescence or early adulthood and after a mood stabilizer has brought their bipolar condition under control, then reporting ADHD symptoms. Rather than argue for an induction sequence, this scenario is more likely to reflect either late-onset ADHD or the patient being unaware of earlier ADHD symptoms.

Turning to the impact of comorbid status, Comparelli et al.^8^ overviewed studies evaluating clinical differences between those with the two conditions as against individuals having one condition alone. Comorbid status was associated with an earlier age of initial psychiatric symptoms and of ADHD symptoms, a poorer developmental history, a greater likelihood of violence, legal problems, rule-breaking behaviours, anxiety, drug and alcohol problems, as well as both a worse mood trajectory and a poorer response to mood-stabilizing medication. In addition, Klassen et al.^9^ overviewed studies indicating that those with both conditions have a lower quality of life and worse overall functioning, and that they present with manic symptoms at an earlier age than those with a bipolar disorder alone, have both a more severe course and more frequent depressive episodes and, whether as a consequence of such factors or not, are less compliant with medication.

Turning to management, clinicians assessing a newly referred patient commonly seek to determine ‘What is the diagnosis?’ Here, as for many other clinical scenarios, the objective should be to determine ‘What are the diagnoses?’ Just as all patients presenting with a depressive condition should be screened for a bipolar disorder, the current overview quantifying the high comorbidity rate argues strongly for all patients diagnosed with a bipolar disorder being screened for ADD or ADHD and, if not identified, being rescreened once the bipolar condition is brought under control as the bipolar condition may have masked its identification.

Comorbid ADHD status may influence response to mood-stabilizing medication, with Strober et al.^14^ reporting that adolescents experiencing mania show a poorer response to lithium if they had ADHD in earlier years. The paucity of such studies argues for similar research in relation to other mood stabilizers.

When both conditions are diagnosed the options are to treat them coterminously or sequentially, with the latter approach being more logical in respecting longstanding concerns about stimulant drugs inducing manic episodes in those with a mood disorder. One approach is to first bring the bipolar condition under control with mood-stabilizing medication (and trying to ensure that such individuals cease any recreational stimulant use) and then, if ADD or ADHD is still distinctive (as it can sometimes no longer be evident or clinically distinct), introduce a prescribed stimulant. An alternative strategy of trialling a psychological program for ADHD before initiating a stimulant drug at that time might also be considered. Such sequencing strategies may be complicated by hospital-based services managing those with a bipolar disorder not readily prescribing stimulant drugs while private practitioners may be less likely to manage those with more severe bipolar disorders.

While many clinicians judge prescribing a stimulant drug to those with a bipolar disorder as risky, evidence presented by Salvi and colleagues^4^ argues against that concern being salient if the patient is taking a mood-stabilizing medication and their bipolar condition is stable or relatively stable. For example, Viktorin et al.^15^ accessed Swedish registries, identified 2307 adults with a bipolar disorder, and prescribed methylphenidate and with participants having their progress reviewed 3 months later. The sample was subdivided into those receiving or not receiving a mood stabilizer. The researchers found no evidence of an increased rate of manic episodes if a mood stabilizer had been prescribed but a distinct increase in those who had the stimulant initiated without participants having been prescribed a mood stabilizer. In an earlier study of 40 adults with a medication stabilized bipolar I or bipolar II disorder and ADHD treated with lisdexamfetamine – and which proved effective for the latter condition – McIntyre et al.^16^ reported no increased induction of any treatment-emergent hypomanic or manic state. Thus, concerns about a stimulant worsening the bipolar disorder can probably be dismissed if there is a mood stabilizer in place and the bipolar disorder is under relative control – and the patient is warned about such a risk. Such a sequencing model is favoured over treating the ADHD with a stimulant first (and so risking a hypo/manic episode) or introducing treatment for both conditions at the same time – when the latter approach may confound interpretation of benefits and any medication side-effects.

We can conclude that the coterminous presence of ADHD and a bipolar disorder is relatively common in clinical practice, warrants pursuit of such a possibility in all patients nominating only one of the two conditions, that underlying mechanisms are likely to be multiple and remain to be established, and that there is strong support for a sequencing management model that seeks to first stabilize the bipolar disorder before prescribing a stimulant medication for the ADHD condition.

## Data Availability

Data sharing is not applicable to this article as no datasets were generated or analysed during the current study.*
